# The clinicopathologic characteristics and prognostic significance of triple-negativity in node-negative breast cancer

**DOI:** 10.1186/1471-2407-8-307

**Published:** 2008-10-23

**Authors:** Jiyoung Rhee, Sae-Won Han, Do-Youn Oh, Jee Hyun Kim, Seock-Ah Im, Wonshik Han, In Ae Park, Dong-Young Noh, Yung-Jue Bang, Tae-You Kim

**Affiliations:** 1 Department of Internal Medicine, Seoul National University Hospital, Seoul, Korea; 2 Department of Internal Medicine, Seoul National University Bundang Hospital, Seongnam, Korea; 3 Department of Surgery, Seoul National University Hospital, Seoul, Korea; 4 Department of Pathology, Seoul National University Hospital, Seoul, Korea; 5 Cancer Research Institute, Seoul National University College of Medicine, Seoul, Korea

## Abstract

**Background:**

Triple-negative (TN) breast cancer, which is defined as being negative for the estrogen receptor (ER), the progesterone receptor (PR), and the human epidermal growth factor receptor 2 (HER-2), represents a subset of breast cancer with different biologic behaviour. We investigated the clinicopathologic characteristics and prognostic indicators of lymph node-negative TN breast cancer.

**Methods:**

Medical records were reviewed from patients with node-negative breast cancer who underwent curative surgery at Seoul National University Hospital between Jan. 2000 and Jun. 2003. Clinicopathologic variables and clinical outcomes were evaluated.

**Results:**

Among 683 patients included, 136 had TN breast cancer and 529 had non-TN breast cancer. TN breast cancer correlated with younger age (< 35 y, *p *= 0.003), and higher histologic and nuclear grade (*p *< 0.001). It also correlated with a molecular profile associated with biological aggressiveness: negative for bcl-2 expression (*p *< 0.001), positive for the epidermal growth factor receptor (*p *= 0.003), and a high level of p53 (*p *< 0.001) and Ki67 expression (*p *< 0.00). The relapse rates during the follow-up period (median, 56.8 months) were 14.7% for TN breast cancer and 6.6% for non-TN breast cancer (*p *= 0.004). Relapse free survival (RFS) was significantly shorter among patients with TN breast cancer compared with those with non-TN breast cancer (4-year RFS rate 85.5% *vs*. 94.2%, respectively; *p *= 0.001). On multivariate analysis, young age, close resection margin, and triple-negativity were independent predictors of shorter RFS.

**Conclusion:**

TN breast cancer had higher relapse rate and more aggressive clinicopathologic characteristics than non-TN in node-negative breast cancer. Thus, TN breast cancer should be integrated into the risk factor analysis for node-negative breast cancer.

## Background

Since 2001, breast cancer has been the most common cancer in women in Korea [1]. While its incidence appears to be levelling off in Western countries, after decades of increasing, it is still high and continues to increase in certain countries where it initially had a low incidence [2].

Early detection of breast cancer and the use of aggressive multimodal treatment have successfully resulted in a decrease in the mortality due to the disease [2]. Prognostic and predictive factors have been widely used in treatment decisions [2]. These factors include: the extent of axillary lymph node involvement, histologic grade, age of the patient, status of hormone receptors (HRs) and human epidermal growth factor receptor 2 (HER2), and involvement of lymphatic or microvascular spaces [2]. Recent studies suggest that breast cancer is a heterogeneous disease and patients with the same diagnostic and clinical prognostic profile can have markedly different clinical outcomes. Therefore, further understanding of the biology of the disease is needed to improve treatment outcome and reduce mortality [2]. Gene-expression profiling has identified five subtypes of breast cancer (luminal A, luminal B, normal breast-like, HER2-overexpression, and basal-like), each of which have a different prognosis[3,4]. The basal-like and HER2+ subtypes have shorter relapse-free and overall survival than the luminal tumours [3-6].

Basal-like breast cancers are often called 'triple-negative' (TN) breast cancer, defined as estrogen receptor-negative, progesterone receptor-negative (i.e., HR-negative), and HER2-negative. Approximately 80% to 90% of TN phenotypic breast cancers are deemed to be basal-like when appropriately tested for immunohistochemical markers and gene expression. Moreover, there is a consistent trend across studies confirming unfavourable clinical outcomes associated with the TN phenotype or basal-like breast cancer [4-14].

Previous studies in Western countries show that TN breast cancer has aggressive clinical and pathologic features, including onset at a young age, advanced stage at diagnosis, high histologic and nuclear grade, high mitotic index, higher frequency of unfavourable histologies, and more distant recurrence [8,10,12,15]. In addition, evidence indicates that the prevalence and clinical outcome of TN breast cancer differs among races [8,15]. Bauer et al. have reported that TN breast cancer is more prevalent among non-Hispanic black compared with other ethnic group, who, when affected with this subtype had the worst survival [8]. Carey et al. also reported that basal-like breast tumours occurred at a higher prevalence among African-American women compared with other racial group [15]. However, there are limited studies of the prevalence, characteristics, and prognosis of TN breast cancer in Asian populations. A recent study of Korean patients indicated that the basal-like subtype, which is positive for one or more of the basal markers and negative for HRs and Her2/neu, was not associated with a poor prognosis. This study also showed that the survival rate associated with the basal-like subtype does not differ from that of other subtypes, with the exception of the Her2/neu-overexpressing subtype, which has the worst survival rate [16]. In contrast, a recent study of breast cancer patients receiving neoadjuvant chemotherapy showed that TN breast cancer was associated with shorter survival than other subtypes, even though it was associated with a higher response rate [11].

The present study was designed to investigate the clinicopathologic characteristics and prognostic significance of TN breast cancer in Koreans.

## Methods

### Patients

Patients who were diagnosed with breast cancer and underwent curative surgery at Seoul National University Hospital between January 2000 and June 2003 were included in the study. The inclusion criteria were: (1) breast cancer with negative lymph nodes on pathological examination; (2) available results of immunohistochemistry for HRs and HER2. Patients who received adjuvant trastuzumab (n = 1) or neoadjuvant chemotherapy (n = 2) were excluded. We retrospectively evaluated each patient's clinicopathologic features, molecular markers and clinical outcome. This study protocol was approved by the Institutional Review Board (IRB) of the Seoul National University Hospital (IRB protocol number H-0809-073-258). Because this study was a retrospective analysis that involved no more than minimal risk for the subjects, the IRB approved our request for the waiver of informed consent.

### Pathological Examination and Immunohistochemistry

Pathological examination included the type of tumour, the tumour stage according to the criteria established by the 6th edition of American Joint Committee on Cancer cancer staging manual, the presence of endovascular or lymphatic tumour emboli, the status of the resection margin, and the histologic and nuclear grade according to the Elston and Ellis modification of the Scarff-Bloom-Richardson grading system [17,18].

Immunohistochemistry was used to test for the expression of the following molecular markers: the estrogen receptor (ER), progesterone receptor (PR), HER2, p53, Ki67, bcl2 and the epidermal growth factor receptor (EGFR). The routinely formalin-fixed, paraffin-embedded tissue blocks were sectioned at 4-μm thickness and then used for immunohistochemistry. Tissue sections were deparaffinized in xylene, rehydrated with graded ethanol, and immersed in Tris-buffered saline. After an antigen-retrieval process, primary antibodies were used as previously described [19]. ER, PR, HER2, p53, Ki67, bcl2 and EGFR expressions were evaluated by the avidin-biotin complex immunohistochemical technique [20]. The primary antibodies were supplied by Novocastra Laboratories Ltd., (New Castle-Upon-Tyne, UK), for HER2, and by the Dako Corporation (Carpinteria, CA, USA) for all of the other markers studied. The dilution factors were as follows: ER (clone 1D5), 1:50 PR (clone PgR636^1^), 1:50; HER2 (clone CB11), 1:200; p53 (clone DO-7A), 1:1200; Ki-67 (clone MIB-1), 1:800; bcl-2 (clone 124), 1:50; and EGFR (clone H11), 1:50. All primary antibodies were mouse monoclonal antibodies. Biotinylated anti-mouse antibody was used as a secondary antibody and streptavidin horseradish peroxidase (Zymed laboratories, San Francisco, CA) methods were used according to the instructions provided by the manufacturer. ER and PR positivity was defined as the presence of 10% or more positively stained nuclei in ten high-power fields. The intensity of HER2 membrane staining was scored as 0, 1+, 2+ or 3+. Tumours with 3+ scores were classified as positive for HER2 overexpression, whereas tumours with 0 or 1+ scores were considered as negative. And tumours with 2+ scores were classified as undetermined [12]. EGFR staining was considered positive if membrane staining was observed. Ki-67 of < 20% and p53 of < 25% were considered low expression.

## Statistical analysis

The comparisons of clinicopathologic variables and patterns of relapse between TN breast cancer and non-TN breast cancer were made using Pearson's χ^2 ^test or Fisher's exact test as appropriate. Two-sided *p *values of < 0.05 were considered statistically significant. The associations between molecular markers and clinicopathologic variables, including TN breast cancer and relapse-free survival (RFS), were analyzed by Kaplan-Meier plots and log-rank tests. The RFS was calculated from the date of surgery to the first detection of disease recurrence. Multivariate analyses were carried out using the Cox regression model. A significance level of 0.05 was used for covariate entry. SPSS for Windows, version 12.0 (SPSS, Inc., Chicago, IL, USA), was used for all statistical analyses.

## Results

### Patient characteristics

A total of 1135 patients were diagnosed with breast cancer and underwent curative surgery. Of these, 683 patients were included in the study. The demographic and clinical characteristics of the patients are summarized in Table 1. Four hundreds and nine patients (59.9%) were ER-positive, 273 patients (40.1%) PR-positive, 175 patients (25.6%) HER2-positive and 123 patients (18%) HER2-undetermined [12]. One hundred and thirty-six patients (19.9%) were TN breast cancer identified as ER-negative, PR-negative, and HER2-negative and 529 patients (77.5%) were non-TN breast cancer indentified as HR-positive or HER2-positive. We excluded 18 patients (2.6%) classified as HRs-negative and HER2-undetermined group from further analyses comparing TN breast cancer and non-TN breast cancer. Two hundreds and eighty-four (41.6%) patients underwent breast-conserving surgery and 237 patients among them received adjuvant radiotherapy to the lesion. Four hundreds and eighteen (61.2%) patients received adjuvant systemic chemotherapy. The median duration of follow-up was 56.8 months (range, 1–89.1 months). Fifty-eight (8.5%) patients had relapses of disease during the follow-up period.

**Table 1:** Demographic and clinical characteristics

Variables	No. of patients (n = 683) (%)
Sex	
M	1 (0.1)
F	682 (99.9)
Age (years)	
Median (range)	47 (22–84)
<35	51 (7.5)
≥35	632 (92.5)
Type of surgery	
BCS	284 (41.6)
Mastectomy	399 (58.4)
Histology	
Invasive ductal carcinoma	605 (88.5)
Invasive mucinous carcinoma	23 (3.4)
Invasive papillary carcinoma	17 (2.5)
Invasive lobular carcinoma	8 (1.2)
Metaplastic carcinoma	7 (1.0)
Medullary carcinoma	5 (0.7)
Tubular carcinoma	5 (0.7)
Invasive cribiriform carcinoma	5 (0.7)
Invasive micropapillary carcinoma	3 (0.4)
Mixed invasive lobular and ductal carcinoma	3 (0.4)
Invasive apocrine carcinoma	2 (0.3)
Pathological tumor size	
<2 cm	399 (58.5)
≥2 cm	284 (41.5)
Adjuvant chemotherapy	
None	262 (38.4)
Yes	418 (61.2)
Unknown	3 (0.4)

BCS, Breast conserving surgery

### Clinicopathologic characteristics of TN and non-TN breast cancer

We compared the clinicopathologic features of TN breast cancer with those of non-TN breast cancer (see Additional file 1). One hundred thirty-six (19.9%) patients had TN breast cancer and 529 (77.5%) had non-TN breast cancer. Compared with non-TN breast cancer, TN breast cancer correlated with younger age (below 35 years, *p *= 0.003), higher histologic and nuclear grade (*p *< 0.001 and *p *< 0.001, respectively), negative staining for bcl2 expression (*p *< 0.001), positive staining for EGFR (*p *= 0.003), and high levels of p53 (*p *< 0.001) and Ki67 expression (*p *< 0.001) (see Additional file 1). Although more patients with TN breast cancer had received adjuvant chemotherapy than those with non-TN breast cancer (*p *< 0.001), a greater percentage of those with TN breast cancer relapsed during the follow-up period (14.7% vs. 6.6%, respectively; *p *= 0.004) (see Additional file 1).

### Clinicopathologic variables associated with RFS

We analyzed the association between clinicopathologic variables and RFS. The results of univariate analyses are summarized in Additional file 2 (see Additional file 2). Younger age (below 35 years), breast-conserving surgery, tumour size larger than 2 cm, the presence of endovascular or lymphatic tumour emboli, a close resection margin (< 3 mm), ER-negativity, TN breast cancer, negative staining for bcl-2 expression, and high levels of Ki67 expression correlated with shorter RFS.

On multivariate analysis, younger age (hazard ratio, 2.880; 95% CI, 1.396 to 5.939, *p *= 0.004), a close resection margin within 3 mm (hazard ratio, 4.495;95% CI, 1.011 to 19.986, *p *= 0.048), and TN breast cancer (hazard ratio, 2.382;95% CI, 1.351 to 4.199, *p *= 0.003) were independently associated with shorter RFS (Table 2).

**Table 2:** Independent prognostic factors influencing relapse free survival (multivariate analysis)

Variables	HR*	95% CI	*p *value
Age (< 35 years)	2.880	1.396–5.939	0.004
Close resection margin < 3 mm	4.495	1.011–19.986	0.048
Triple-negative	2.382	1.351–4.199	0.003

HR, hazard ratio; CI, confidence interval.

*Adjusted for age, type of surgery, tumour size, endovascular or lymphatic tumour emboli, status of resection margin, triple negative breast cancer, bcl2, and Ki67

### Patterns of relapse in TN breast cancer

**Figure 1:**
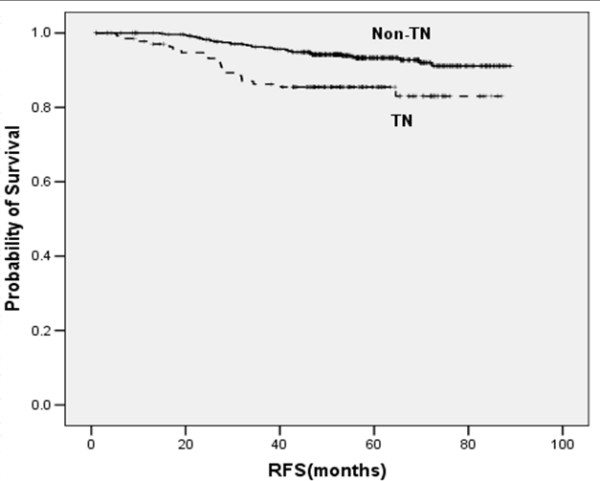
Kaplan-Meier plot of RFS according to triple negative (TN) phenotype.

**Figure 2:**
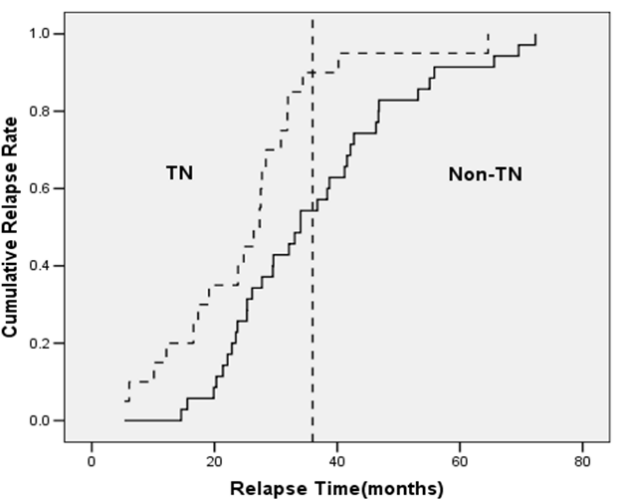
**Kaplan-Meier plot of cumulative relapse rate among patients with relapses.** TN = triple negative breast cancer.

**Figure 3:**
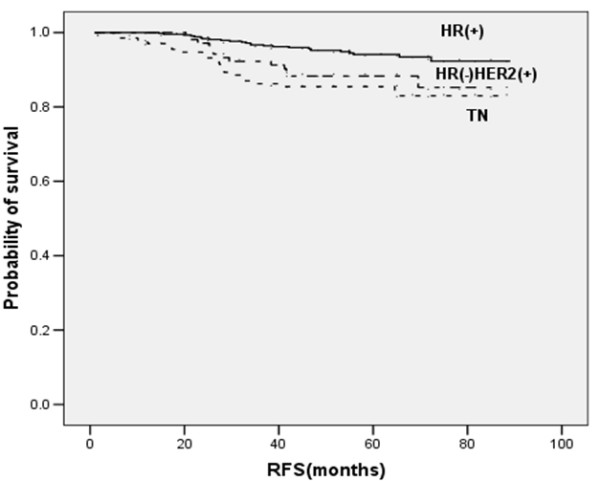
Kaplan-Meier plot of RFS according to HR and HER2 status.

The 4-year RFS rates in patients with TN breast cancer and non-TN breast cancer were 85.5% and 94.2%, respectively (*p *= 0.001) (Figure 1). Eighteen patients (90.0%) with relapsed TN breast cancer had their relapses within 3 years after surgery, whereas 19 patients (57.3%) with relapsed non-TN breast cancer had relapses within 3 year after surgery (*p *= 0.007) (Figure 2). The distribution of the sites of recurrence (distant, locoregional, or contralateral breast) was not statistically different between TN and non-TN breast cancer (*p *= 0.968). TN breast cancer patients who were younger had shorter RFS than those without these features (*p *= 0.028). Patients with TN disease also had shorter RFS than patients who were HR-positive (*p *< 0.001) or HR-negative/HER2 -positive (*p *= 0.384) (Figure 3).

## Discussion

The molecular classification of breast cancer has revealed the heterogeneity of the disease with respect to prognosis and response to therapy. Among the subgroups of breast cancer, TN breast cancer is particularly feared because it is associated with a poor clinical outcome and it has no specific systemic treatment [10-13]. However, clinical data on TN breast cancer in Asian populations are limited. Thus, we investigated the clinicopathologic features and the prognostic indicators of lymph-node negative, TN breast cancer in Koreans.

In the present study, 19.9% (136/683) of the included patients had TN breast cancer. Carey et al. found that the prevalence of the TN subtype among patients with breast cancer in US was 26.4%; among non-African American patients with breast cancer this prevalence was 23% [15]. Bauer et al. reported that in US, the prevalence of TN breast cancer among patients with all forms of breast cancer was 12.4% and that this prevalence was highest among non-Hispanic black patients with breast cancer, at 24.6% [8]. Previous studies among Asian women have reported more than 30% of breast cancer was the TN subtype [11,16]. While the prevalence of TN breast cancer in our study (19.9%) was lower than in these other studies, the prevalence among Koreans may not actually be lower. The lower prevalence in our study may be the result of including only node-negative patients in combination with the association of TN breast cancer with advanced stage and, thus, node-positive status.

In the current study, TN breast cancer was associated with younger age, higher histologic and nuclear grade, negative staining for bcl-2, positive staining for EGFR, and high levels of p53 and Ki67 expression. These characteristics are known to be markers of biologic aggressiveness and poor prognosis in breast cancer [18,21-25]. Our observation that TN breast cancer has a shorter RFS than non-TN breast cancer in lymph-node negative cancer is consistent with most other studies [8-10,12,14,19]. We also found that TN breast cancer was an independent prognostic factor for shorter RFS. These results indicate that the prognosis of TN breast cancer in Korean populations does not differ from that in Western countries.

In the current study, most of the relapses in TN breast cancer occurred within the first 3 years, in contrast to non-TN breast cancer. This finding reflects the aggressiveness of TN breast cancer and is consistent with previously reported results, such as those from the study by Dent et al [10]. They reported that the risk of recurrence declined rapidly after 4 years and no recurrences occurred after 8 years. Rakha et al. reported that the only prognostic marker among the TN breast cancer in the lymph node-negative subgroup was the basal phenotype, defined as the expression of CK5/6 or CK14 [9]. These results suggest the possibility of sub-classifications of TN breast cancer and the necessity for further study. And patients with TN breast cancer had shorter RFS than patients who were HR-positive or HR-negative/HER-2 positive. Considering the high proportion of HER2-positive patients among HR-negative patients (39.5%) in this study and the expected efficacy of adjuvant trastuzumab, it is reasonable to separate TN breast cancer from HR-negative breast cancer in planning treatment [27-29].

The current study has a number of limitations. Some patient records lacked the results of immunohistochemical analyses for biologic markers other than HR and HER2. The result of HER2 fluorescence in situ hybridization in the primary tumour was not available in the majority of patients. In the present study, HER2 0 or 1+ was classified as HER2-negative for clarifying TN breast cancer although a previous study showed that clinical outcome of TN breast cancer was not significantly different whether HER2 2+ patients were classified as HER2-negative or HER2-positive [12]. Eighteen patients (14.6%) of HR-negative patients were classified as HER2-undetermined group. There is lack of consensus regarding the definition of basal-like breast cancer and TN breast cancer. However, in spite of different classifications, there is a consistent result across all studies suggesting the aggressive clinicopathologic and biologic features of TN breast cancer and basal-like breast cancer [3-12]. Another limitation is the short duration of follow-up which makes the overall survival analysis unfeasible. In conclusion, TN breast cancer, defined by negative HR and HER2 status, was associated with more aggressive clinicopathologic features and molecular markers and with shorter RFS. We confirmed TN breast cancer was a significant prognostic factor in lymph-node negative breast cancer in Koreans. Thus, identifying this subtype should be integrated into risk factor analysis for node-negative breast cancer.

Lately, some studies have reported that the phenotypical and molecular features of *BRCA1*-associated breast cancers are sporadically shared by TN breast cancers [29-31]. These findings suggest that the defect in the DNA-repair pathways characteristic of *BRCA1*-related cancers may also occur in TN breast cancers and this molecular defect may be more specifically targeted [32-34]. On the basis of previous data, further studies are needed to define breast cancer subtypes in greater detail and to develop and assess specifically targeted therapies.

## Conclusion

TN breast cancer was associated with more aggressive clinicopathologic features and molecular markers and with shorter RFS. TN breast cancer was a significant prognostic factor in lymph-node negative breast cancer in Koreans.

## Competing interests

The authors declare that they have no competing interests.

## Authors' contributions

JR collected the data, performed the statistical analysis and drafted the manuscript. TYK designed the concept of this study, performed the statistical analysis with interpretation and approved the final manuscript. SWH, DYO, JHK, SAI, TYK, and YJB performed the chemotherapy for patients and revised the manuscript. WH and DYN performed operation and treatment coordination. IAP carried out the immunoassays and pathologic examinations. All authors read and approved the final manuscript.

## Pre-publication history

The pre-publication history for this paper can be accessed here:



## Supplementary Material

Additional file 1 **Comparison between TN and non-TN breast cancer according to clinicopathologic features.** Click here for file

Additional file 2 **The correlation of clinicopathologic variables and molecular markers with relapse free survival (univariate analysis).** Click here for file
